# A Rare Clinical Presentation of Intraoral Darier's Disease

**DOI:** 10.1155/2011/181728

**Published:** 2011-09-08

**Authors:** K. G. D. Manoja, B. S. M. S. Siriwardena, P. R. Jayasooriya, D. J. L. Siriwardane, W. M. Tilakaratne

**Affiliations:** ^1^Department of Oral Pathology, Faculty of Dental Sciences, University of Peradeniya, 20400 Peradeniya, Sri Lanka; ^2^Military Hospital, Colombo, Sri Lanka

## Abstract

Darier's disease, also known as keratosis follicularis or dyskeratosis follicularis, is a rare disorder of keratinization. It is an autosomal dominant genodermatosis with high penetrance and variable expressivity. Its manifestation appears as hyperkeratotic papules primarily affecting seborrheic areas on the head, neck, thorax, and less frequently the oral mucosa. When oral manifestations are present, the palatal and alveolar mucosae are primarily affected. They usually asymptomatic and are discovered in routine dental examination. Histologically, the lesions present as suprabasal clefts in the epithelium with acantholytic and dyskeratotic cells represented by “corps ronds and grains.” This paper reports a case of an adult male patient who presented with painful whitish lesions on buccal mucosa with crusty lips as the only clinical sign of Darier's disease. As this patient did not have skin lesions or family history, an intraoral biopsy confirmed the diagnosis of Darier's disease by a multidisciplinary team.

## 1. Introduction

Darier's disease or keratosis follicularis is a rare autosomal dominant genodermatosis, which is characterized by greasy, crusted, keratotic, yellow brown warty papules and plaques particularly over seborrhoeic areas. Although this is a genetically transmitted disease according to a larger series, about 47% of patient had no clear family history, presumably of incomplete penetrance [1]. The disease is caused by mutations in the ATP 2A gene, which encodes the sarco/endoplasmic reticulum Ca^2+^ ATPase [1]. This disease was first described by Prince Marrow in 1886 and simultaneously by Darrier and White in 1889, independently. In 1917, the first case with oral manifestation was reported by Reenstierna [2]. 

The prevalence of this disorder in the population is 1 : 100,000. The sex incidence is equal, although the males appear to be more severely affected than females [2–4]. The oral mucosa is affected in 50% of the cases [4] and lesions are usually asymptomatic and discovered during routine dental examination [5, 6]. Lesions are represented by multiple firm papules with normal, whitish, or reddish color, primarily affecting the palatal and alveolar mucosa. Initially, papules are reddish and may coalesce, forming crusts that may be ulcerated. Histologically, the lesions present as suprabasal clefts in the epithelium with acantholysis and dyskeratotic cells present as “corps ronds” and “corps grains.” “Corps ronds” are larger structures usually present in the granular layer and consists of an irregular eccentric and sometimes pyknotic nuclei [7]. Precipitating factors include heat and humidity, mechanical trauma like friction, sunlight, and secondary bacterial infections [8]. Associated anomalies have been described in the literature, including mental retardation and psychosis [6]. This paper reports a case of Darier's disease, within the oral mucosa without skin manifestations.

## 2. Case Report

**Figure 1:**
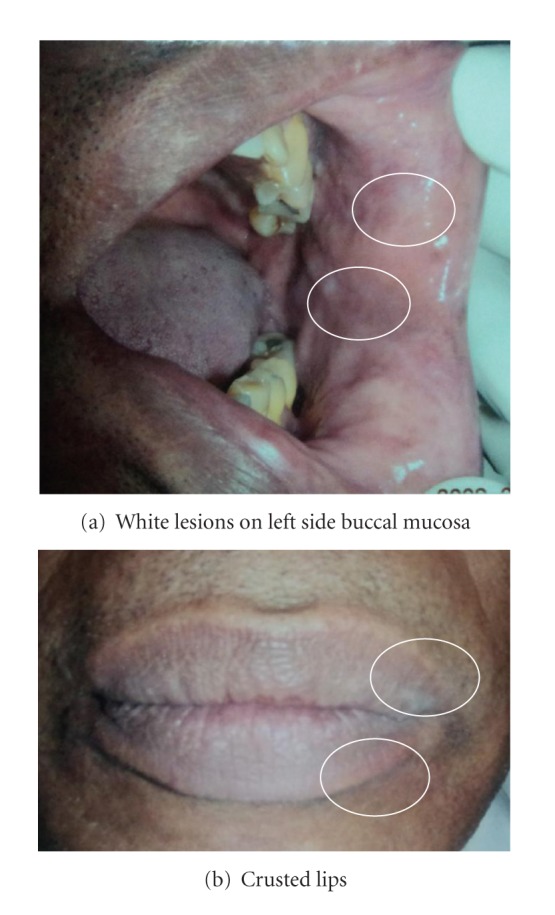
Clinical presentation.

Fifty-two-year-old male patient presented to the Military Hospital, Colombo with whitish lesions on both buccal mucosae and crusty lips (Figures 1(a) and 1(b)) for two years duration. Initially, the patient experienced uncomfortable feeling and dryness of his mouth. The symptoms were exaggerated on hot sunny days and then after a few days lips got crusted and fissured. Gradually, the condition got worse and the tenderness over his outer lip margin was aggravated. Considering his signs and symptoms, clinicians arrived at a differential diagnosis including Darier's disease and Hailey-Hailey disease. 

**Figure 2:**
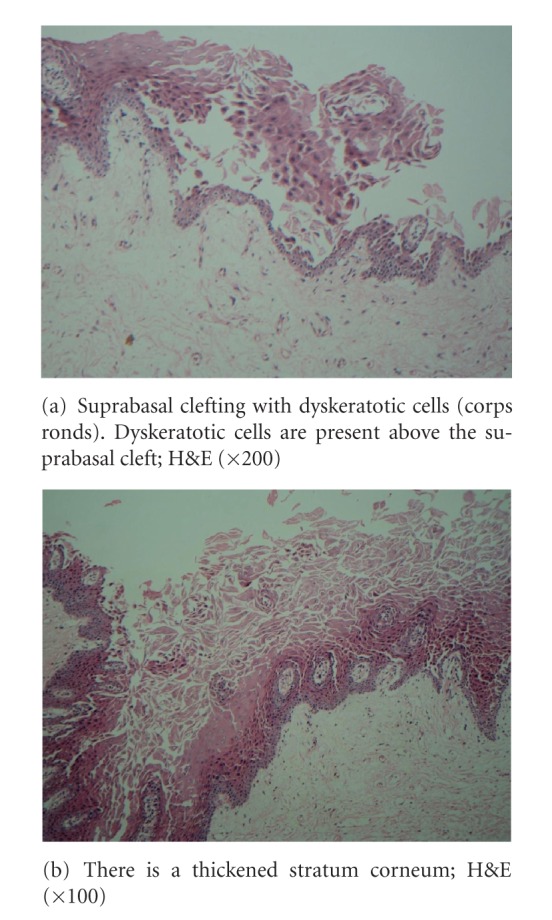
Histopathology.

The patient was on antidiabetic drugs and also having gastric ulcers. He works as a clerk. He was a smoker and a social drinker. The family history was not contributory. Extraorally, the patient showed no recognizable signs of the disease. Intraorally the whole oral mucosa was altered. Multiple asymptomatic coalesced papules with rough texture on palpation were observed in the vermillion border. Lips were dried and crusted. The patient was a denture wearer. Histopathologically, the biopsy revealed the presence of suprabasal splits in the epithelium with acantholytic and dyskeratotic cells (Figures 2(a) and 2(b)) observed as round and granular corpuscles and was reported as keratosis follicularis by a multidisciplinary team. Mouth rinse with antiseptic solutions was prescribed to improve oral hygiene and treated with topical steroid applications and vitamin A supplements.

## 3. Discussion

Darier's disease is an autosomal dominant disease with high penetrance and variable expressivity. Although it is an inherited disease, 47% of the patients with Darier's disease do not have a family history [8]. Absence of family history could also be attributed to the fact that mild forms of the disease have not been recognized among the family members. Mutations in the ATP2A2 gene found on chromosome 12q, which encodes for a sarco/endoplasmic reticulum calcium ATPase pump (SERCA2) type 2 isoform, are the cause of the disease. Ca^2+^ ATPases are the key actors in the regulation of calcium in eukaryotic cells and are thus essential to the correct functioning of the cell machinery [9]. Ca^2+^ ATPase transport Ca^2+^ from the cytosol back to the endoplasmic reticulum lumen hence mediate stability and adhesion of desmosomes. The mutations in this gene affect Ca^2+^ homeostasis and result in abnormality in desmosomal stability and adhesion [10].

Histologically, Darier's disease is characterized by acantholysis which forms suprabasal clefts and also formation of “corps ronds and grains” superficially. Corps ronds are usually present in the granular cell layer and show central large round dyskeratotic basophilic masses surrounded by a clear halo-like zone. Darier's disease must be distinguished histologically from other acantholytic dyskeratoses, such as Hailey-Hailey disease (familial benign pemphigus) and Grover's disease (transient acantholytic dermatosis). In Hailey-Hailey disease, acantholysis is incomplete, causing the well known “dilapidated brick wall” appearance of the lower epidermis [11]. The clinical characteristics of those diseases are different from those of Darier's disease.

Oral lesions are detected in approximately 15% of the patients, and they appear as white papules with a central depression [12]. Here, we report a rare clinical presentation which is confined to the oral cavity. This patient had no cutaneous involvement of the trunk and because of this his diagnosis was not initially suspected. According to the literature, more than 113 familial and sporadic mutations in *ATP2A2* have been identified in the disease. However, the attempts at genotype-phenotype correlation have not been successful. Family members with confirmed identical *ATP2A2* mutations can exhibit differences in the clinical severity of disease, suggesting that other genes or environmental factors affect the expression of keratosis follicularis [13, 14]. The present patient had white patches on perioral region mostly on the lips. Although this disease is inherited, novel mutations of the gene can also pass to next generation causing isolated cases without family history as our patient [9]. 

This disease is mostly affected in 4-5th decades, and our case also belongs to this category. The affected patient with oral lesions show dried, crusted, itchy lesions on seborrheic areas, and similarly this case also presented with crusted lips. Intraoral lesions are usually whitish and show variable consistency; similarly, this patient also had identical clinical features.

Although the present case is not a severe form of Darier's disease, most patients with severe form of Darier's disease should receive genetic counseling, including information on the inherited condition and risk of transmission to offspring.

Since some conditions are asymptomatic, dental surgeons may diagnose the oral manifestations of this disease in routine examination. Biopsy is necessary to arrive at definitive diagnosis. Patients should be referred for dermatological examination and should be informed about the possible complications like bad odors, caries, and secondary infections. Psychiatric opinion should follow in more severe cases. Therefore, it is important to ensure multidisciplinary approach in the management of patients with Darier's disease.
